# Supplementary figures and images for: Introducing platform surface interior angle (PSIA) and its role in flake formation, size and shape
Source: PLoS One. 2020 Nov 18;15(11):e0241714. doi: 10.1371/journal.pone.0241714 (PMC7673556; doi:10.1371/journal.pone.0241714)

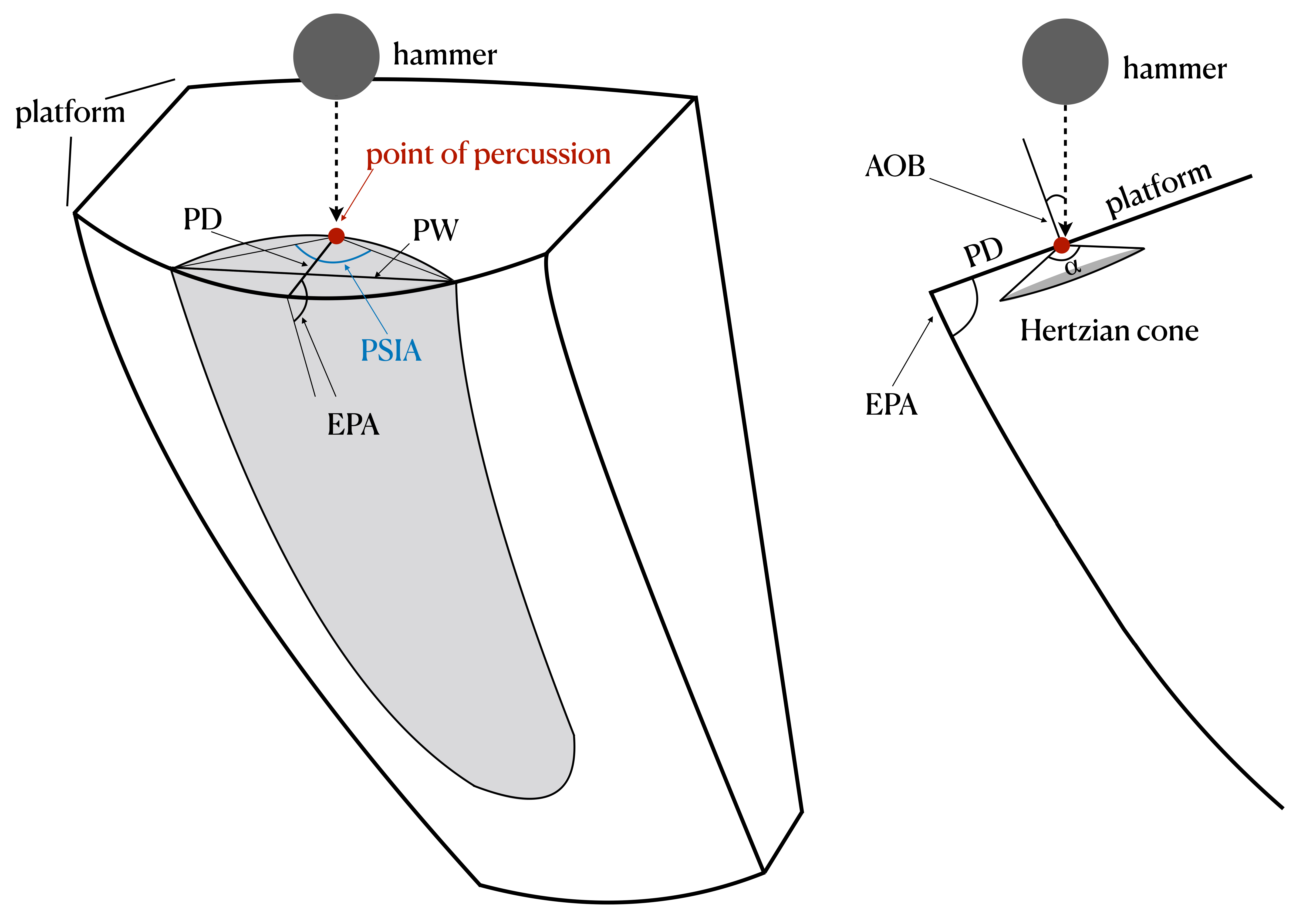

Supplement: S2 File — (ZIP) [file pone.0241714.s002.zip › figure_01.png]

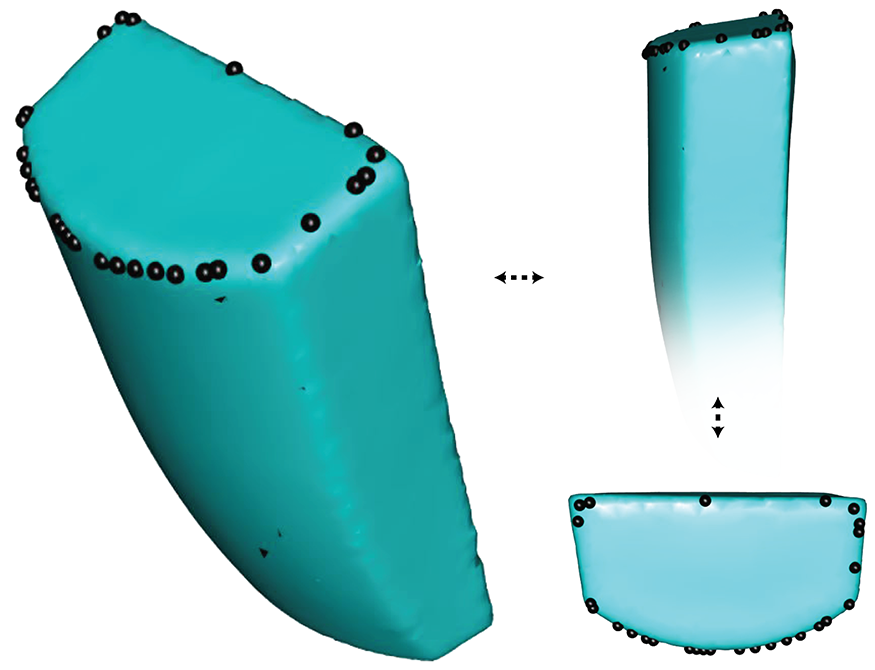

Supplement: S2 File — (ZIP) [file pone.0241714.s002.zip › Figure_2a.png]
