# Supplementary material for: Introducing platform surface interior angle (PSIA) and its role in flake formation, size and shape
Source: PLoS One. 2020 Nov 18;15(11):e0241714. doi: 10.1371/journal.pone.0241714 (PMC7673556; doi:10.1371/journal.pone.0241714)
Supplement: Attachment — Submitted filename: Comments.pdf [file pone.0241714.s003.pdf]

Comments on PONE-D-20-16801

In the present manuscript, the linkage between fracture mechanics and the results obtained from controlled experiments is explored. By documenting the influence of Hertzian cone formation, the authors found that the platform width is a function of the Hertzian cone constant angle and the geometry of the platform edge. In the referee's opinion, the formulation and method of this paper should be right, but the following revision should be carefully made:

1. The drawings in the paper are bad, please improve them. For example, the present setup presented in Fig.1 is unclear.

2. There is something wrong with the citation in the paper, for example, no issue number for Reference 17.

3. There are plenty of improper expressions in the manuscript, for example,

"Increasing either increases flake size, but the relationship between the two is geometric such that at higher values of EPA changes in PD have a greater effect on flake size."

"The EPA-PD model of flake formation, however, is constrained in what it can explain."

"In particular, we start with the principle that the Hertzian cone, the angle of which is known from fracture mechanics to be a constant for a given raw material, has a measurable impact on flake formation."

"While the size of the Hertzian cone is dependent on variables such as the indenter's radius, impact velocity and fracture toughness of the brittle solid, the cone angle

remains unchanged”

“This dataset has the advantage that a number of potentially important variables are either controlled for or were measured.”

“In the results presented below, this angle is referred to as the estimated PSIA to indicate that is is not directly measured from the flakes themselves.”

Please check the manuscript carefully and improve them.

4. More explanation about the EPA-PD model and PSIA will be more useful for readers of the present paper.

5. The reviewer suggests the authors to add detailed information about the solution procedure in the present paper.

6. Whether the present model is related to contact behavior of an indenter on a substrate? If so, some related references should be helpful, for example, J. Appl. Mech.-Trans. ASME. 2015. 82(4): 041008. Int. J. Solids Struct. 2013. 50(7): 1108-1119. Int. J. Mech. Sci. 2019. 151: 410-423.
