# Supplementary material for: Introducing platform surface interior angle (PSIA) and its role in flake formation, size and shape
Source: PLoS One. 2020 Nov 18;15(11):e0241714. doi: 10.1371/journal.pone.0241714 (PMC7673556; doi:10.1371/journal.pone.0241714)
Supplement: Attachment — Submitted filename: Response Document.pdf [file pone.0241714.s004.pdf]

Response Document

Reviewer #1: The paper by McPherron et al introduces PSIA- Platform Surface Interior Angle as a new quantifiable variable to understand fracture mechanics in flake formation, shape, and size variability through statistical modelling. This study adds a constant to the EPA-DP model and the study of fracture mechanics to explain flake variability. Though there is a need for further testing and data, which is clearly stated by the authors, the paper sets the principles and methodology to achieve it and expand it towards a comprehension of flake formation. I believe it will be a reference for a growing research field and particularly important towards the development of a general model for understanding technological variability.

Only minor revisions are recommended: Page 5 (103-105): the word “majority” can be subjective. Perhaps following the argument with supporting references would enrich the paragraph.
Whew. Not sure how to address this one. We agree that it is a subjective statement without citation, but we also think that it would be difficult to develop this point without at least an additional paragraph where we would have to call out individual studies in a somewhat negative way. This, we think, would distract from our paper. What we have done is reworded a bit and added a citation to Speth 1972 where he makes the same point decades ago.

Page 8 (179-184): Though the paper does not centre on raw material variability, the description criteria for the three assemblages used in the study should be homogenous. In materials and methods, the description of Dibble glass data and Campagne data refer to the raw material used. For the MPI data, this information is missing.
We added that the flakes are made from flint.

Page 8 (200): I assume the filtered Dibble glass data is represented by 49 samples referred on page 11 - 288. To make it easier for the reader to follow the results, the number of samples should be included earlier in the description of materials and methods.
There is a confusion here and we modified the text to make this more clear. First, we restate the Dibble glass sample size (142) when discussing the filtering. Second, no, the 49 figure is a subsample of the 142. We now call this a subsample and we restate the sample sizes.

Figure 10: it is recommended to add labels in figure 10. Though the same labels are present in figure 9, it makes it easier for the reader to have the corresponding information in each graph or figure.
Done.

Reviewer #2

Comments on PONE-D-20-16801

In the present manuscript, the linkage between fracture mechanics and the results obtained from controlled experiments is explored. By documenting the influence of

Hertzian cone formation, the authors found that the platform width is a function of the Hertzian cone constant angle and the geometry of the platform edge. In the referee's opinion, the formulation and method of this paper should be right, but the following revision should be carefully made:

1. The drawings in the paper are bad, please improve them. For example, the present setup presented in Fig.1 is unclear.

   We redrew the figure to make it more clear and slightly better in appearance. We note, however, that this figure is in keeping with similar figures in the literature.

2. There is something wrong with the citation in the paper, for example, no issue number for Reference 17.

   This particular publication does not have an issue number. However, pages were missing, and we corrected that. We also went through all other citations and checked them for accuracy and missing information. Several were corrected. We also updated our citation to R.

3. There are plenty of improper expressions in the manuscript, for example,

   "Increasing either increases flake size, but the relationship between the two is geometric such that at higher values of EPA changes in PD have a greater effect on flake size."

   We modified this sentence to increase clarity.

   "The EPA-PD model of flake formation, however, is constrained in what it can explain."

   We think this sentence is clear, especially in the context of what follows.

   "In particular, we start with the principle that the Hertzian cone, the angle of which is known from fracture mechanics to be a constant for a given raw material, has a measurable impact on flake formation."

   We struggle to find a way to say this more clearly.

   "While the size of the Hertzian cone is dependent on variables such as the indenter's radius, impact velocity and fracture toughness of the brittle solid, the cone angle

remains unchanged”

We modified this sentence slightly.

“This dataset has the advantage that a number of potentially important variables are either controlled for or were measured.”

We struggle to find a way to say this more clearly.

“In the results presented below, this angle is referred to as the estimated PSIA to indicate that is is not directly measured from the flakes themselves.”

Fixed.

Please check the manuscript carefully and improve them. **We have done this.**

4. More explanation about the EPA-PD model and PSIA will be more useful for

   readers of the present paper.

   We are not sure what else we can say about the EPA-PD model, and we have provided extensive citations to the original papers defining this model plus papers applying it. And given that we are introducing PSIA for the first time and that the paper is entirely about PSIA, we are also not sure what more explanation we can give. We want to emphasize to the reviewer that we do not understand the mechanics behind these models as well as we would like. This paper tries to tie PSIA to Hertzian cone formation, but exactly how that happens is unclear (not just to us). These will be things that additional research can continue to clarify.

5. The reviewer suggests the authors to add detailed information about the solution

   procedure in the present paper.

   We are not sure what a solution procedure is, and so we Googled it. If we understand correctly from the examples we found, this is well beyond our expertise to do. We also doubt we have a sufficient understanding of the mechanisms involved at this point to do such a thing. While not exactly the same thing, we do note that all of our code and all of our data are included with this paper.

6. Whether the present model is related to contact behavior of an indenter on a

   substrate? If so, some related references should be helpful, for example, J. Appl.

   Mech.-Trans. ASME. 2015. 82(4): 041008. Int. J. Solids Struct. 2013. 50(7): 1108-1119. Int. J. Mech. Sci. 2019.

   151: 410-423.

We thank the reviewer for drawing our attention to these papers. As we further investigate different strike conditions, we think this literature could be valuable. It certainly points to issues we will need to consider. For the present paper, however, we think that this discussion on our part would be too speculative at this time and draw away from our main point.
